# Characterization of rapid weight gain phenotype in children with narcolepsy

**DOI:** 10.1111/cns.13811

**Published:** 2022-02-25

**Authors:** Min Zhang, Marine Thieux, Clara Odilia Inocente, Noemie Vieux, Laura Arvis, Carine Villanueva, Jian‐Sheng Lin, Sabine Plancoulaine, Aurore Guyon, Patricia Franco

**Affiliations:** ^1^ Integrative Physiology of the Brain Arousal Systems CRNL INSERM U1028 CNRS UMR5292 Université Claude Bernard Lyon 1 Lyon France; ^2^ Pediatric Sleep Unit Hôpital Femme Mère Enfant Hospices Civils de Lyon & National Reference Center for Narcolepsy Université Claude Bernard Lyon 1 Lyon France; ^3^ Pediatric Endocrinology Unit Hôpital Femme Mère Enfant Hospices Civils de Lyon Université Claude Bernard Lyon 1 Lyon France; ^4^ Université de Paris CRESS INSERM INRAE Paris France

**Keywords:** children, narcolepsy, obesity, rapid weight gain, sleep

## Abstract

**Objectives:**

To characterize the rapid weight gain (RWG) phenotype associated with the onset of childhood narcolepsy and to determine whether it could constitute a marker of severity of the disease.

**Methods:**

RWG was defined using the BMI z‐score slope reported to one year (>0.67 SD) from symptom onset to disease diagnosis. We compared the clinical, metabolic, and sleep characteristics between patients with or without RWG at diagnosis. Pharmacological management, anthropometric, and clinical progression were also evaluated during the follow‐up.

**Results:**

A total of 84 de novo narcoleptic pediatric patients were included; their median age at diagnosis was 12.0 years; 59.5% boys, 90.5% cataplexy, and 98.7% HLA‐DQB1*06:02, 57% had RWG profile. RWG patients were younger at diagnosis than non‐RWG patients, despite a shorter diagnostic delay. They had a higher BMI z‐score and a higher prevalence of obesity at diagnosis, but not at symptom onset, and higher adapted Epworth Sleepiness Scale and Insomnia Severity Index scores than non‐RWG patients. No differences on nocturnal polysomnography and multiple sleep latency tests were found between groups at disease diagnosis. After a median follow‐up of 5 years, RWG patients still had a higher BMI z‐score and a higher prevalence of obesity despite benefiting from the same therapeutic management and displaying improvement in sleepiness and school difficulties.

**Conclusions:**

Narcoleptic RWG patients were younger, sleepier, and the prevalence of obesity was higher at diagnosis despite a shorter diagnostic delay than that of non‐RWG patients. These patients had also a higher risk of developing a long‐term obesity, despite a positive progression of their narcoleptic symptoms. RGW could then represent a maker of a more severe phenotype of childhood narcolepsy, which should inspire a prompt and more offensive management to prevent obesity and its complications.

## Graphical abstract

To characterize the rapid weight gain (RWG) phenotype of childhood narcolepsy and to determine whether it could constitute a marker of severity. Compared to non‐RWG patients, RWG narcoleptic children had shorter diagnosis delay, were younger, more obese, sleepier with more dyssomnia at diagnosis, likely reflecting severer autoimmune attack, but were also more obese at follow‐up. RGW could represent a maker of a more severe phenotype of childhood narcolepsy.

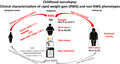

## 1 INTRODUCTION

Narcolepsy is a rare neurological disorder characterized by excessive daytime sleepiness (EDS) and abnormal rapid eye movement (REM) sleep manifestations including cataplexy, hypnagogic hallucinations, and sleep paralysis.^1^ For more than half of narcoleptic patients, symptoms appear before the age of 18 years.^2^ The human leukocyte antigen (HLA) DQB1*0602 genotype is closely associated with narcolepsy.^3^ It is generally believed that a hypocretin neuronal loss in the lateral hypothalamus^4^ constitutes the direct cause of narcolepsy with cataplexy (NC). As the human leukocyte antigen (HLA) DQB1*0602 genotype is closely associated with narcolepsy,^3^ this disappearance of hypocretin cells is generally imputed to autoimmune mechanisms.^5^, ^6^ NC with cerebrospinal fluid (CSF) hypocretin‐1 levels ≤110 ng/ml is also called narcolepsy type 1 or hypocretin‐deficient (NT1). Hypocretins are not only involved in sleep‐wake control but have also been involved in feeding behavior and energy balance regulation.^7^ More than 50% of narcoleptic children are obese/overweight^8^, ^9^ (higher prevalence than among adult patients),^10^ and more than 80% of them experience a rapid weight gain (RWG) soon after symptom onset,^11^ which has drawn increasing clinical attention.^12^ The clinical picture and mechanisms involved in RWG remain poorly illustrated, even though the rapid body mass index (BMI) increment in NT1 children has been attributed to a decreased energy expenditure at symptom onset.^13^

Notably, the characteristics of the patients who developed RGW have never been addressed. Although Ponziani et al. have analyzed the anthropometric characteristics of 30 NT1 children over a 2‐year period before NT1 onset and have found a weight gain starting close to NT1 onset,^12^ only the comparison between narcoleptic children with or without obesity has been made. It is noteworthy that some narcoleptic patients have developed obesity without RWG, and that some patients are not obese at diagnosis despite having experienced RWG. The present study sought to determine whether narcoleptic children with/without RWG after symptom onset had distinct clinical and polysomnographic characteristics in order to assess whether this RGW criteria could constitute a marker of the severity of the disease. Firstly, we compared the clinical, anthropometric, endocrinological, metabolic, and sleep characteristics at diagnosis between narcoleptic patients with and without RWG. Secondly, we evaluated over a follow‐up their pharmacological management, clinical and anthropometric changes, and academic achievements.

## 2 METHODS

### 2.1 Patients

In this retrospective study, data regarding de novo pediatric narcoleptic patients were collected from the pediatric sleep unit of the *Hôpital Femme Mère Enfant* (*Hospices Civils de Lyon*, Lyon, France) between 2008 and 2019. No patient was treated before being referred to the mentioned sleep center. All patients and their parents were informed of the research procedure, and parents signed a written informed consent. This study was approved by the local ethic committee and by the *Commission nationale de l'informatique et des libertés* (CNIL: French national data protection agency, CNIL register n ° 19–087).

### 2.2 Diagnostic procedure

Patients filled up a sleep log during the 15 days preceding the sleep laboratory evaluation. Each patient underwent nocturnal polysomnography (PSG) followed by the multiple sleep latency test (MSLT) with 4 or 5 nap opportunities; this assessment was stopped after 20 min if no sleep occurred, and after 15‐min asleep if sleep occurred.^14^ The polysomnography included eight electroencephalogram (EEG) electrodes, two electrooculograms (EOG), and surface electromyograms (EMG); the latter were located on the mentalis muscle, the left and right anterior tibialis muscles, and the oral thermistor, thoracic, and abdominal belts; electrocardiogram and nasal pressure were recorded using cannulae.^14^ Sleep stages, arousals, and respiratory events were scored visually according to standard pediatric criteria by an experienced sleep specialist (PF).^14^

### 2.3 Criteria for idiopathic narcolepsy diagnosis

The criteria for the diagnosis of idiopathic narcolepsy^15^ were: (1) complaints of excessive daytime sleepiness for at least 3 months, (2) symptoms not better explained by other medical or psychiatric disorders, (3) absence of secondary narcolepsy, (4) presence or absence of clear‐cut cataplexy, and (5) mean sleep latency ≤8 min and ≥2 sleep onset REM periods (SOREMPs) on MSLT or during the previous night PSG. However, due to the occurrence of borderline MSLT results in pediatrics (mean sleep latency ≥8 min or only one SOREMP), a recent study has defined the MSLT criteria in childhood NT1 as a mean sleep latency ≤8.2 min or ≥2 SOREMPs on MSLT including previous night PSG.^16^ HLA‐DQB1*0602 genotyping was determined for all patients, and CSF hypocretin levels were measured for some patients. Brain magnetic resonance imaging was carried out to exclude secondary narcolepsy.

### 2.4 Questionnaires

During the hospitalization for the diagnosis of narcolepsy, daytime sleepiness was evaluated using the adapted Epworth Sleepiness Scale (AESS),^17^ for which a score >10 is considered pathological. The severity of cataplexy (1=moderate weakness, for example, head drop or jaw opening; 2=can maintain posture with external support; 3=loss of posture and falls to the ground)^18^ and the frequency of cataplexy attacks (0: <1 attack/year; 1: ≥1 attack/year; 2: ≥1 attack/month, 3: ≥1 attack/week; 4: ≥1 attack/day)^19^ were evaluated. Depressive feelings were evaluated using the Children's Depression Inventory (CDI),^20^ for which a score ≥16 is considered pathological. Attention‐deficit/hyperactivity disorder symptoms were scored by parents using the revised version of the Conners Parents Rating Scale,^21^ for which a score ≥65 or ≥75 corresponds to moderate or severe symptoms, respectively. Insomnia was evaluated using the Insomnia Severity Index (ISI),^22^ for which a score ≥10 is considered pathological. The severity of the disease was assessed using the Narcolepsy Severity Scale (NSS),^23^ a self‐administered 15‐item scale evaluating the severity, frequency, and impact of five narcolepsy symptoms (EDS, cataplexy, hallucinations, sleep paralysis, and disrupted nighttime), the item regarding driving was ignored, and higher scores indicating the more severe symptoms.

### 2.5 Anthropometric measurements

Height and weight at diagnosis were measured in upright position during the hospitalization and those at symptom onset were collected from the *carnet de santé* (record of all anthropometric, vaccinal, and health‐related data about a child from birth by a pediatrician). Anthropometric data at a time‐point close to symptom onset were considered for analysis when there were no available data at symptom onset. Symptom onset was defined as the occurrence of sleepiness because it appears before other symptoms, such as cataplexy in the population studied herein. The time of PSG evaluation corresponded to the diagnostic time. The BMI was computed at symptom onset and disease diagnosis. Overweight and obesity were defined as BMI was > +1 standard deviation (SD) and > +2 SD for sex and age in children >5 years old, respectively, and as BMI was > +2 SD and > +3 SD for sex and age in children ≤5 years old, respectively, in accordance with the criteria recommended by the World Health Organization (WHO).^24^ BMI *z*‐score (i.e., weight adjusted for height, sex, and age) was also computed at symptom onset and disease diagnosis.^25^ No underweight child was included in our study. The RWG phenotype was defined by a BMI z‐score slope reported to one year (difference between the BMI z‐score at symptom onset and at disease diagnosis divided by the diagnostic delay expressed in years) >0.67 SD; the non‐RGW phenotype was defined by a BMI z‐score slope reported to one year ≤0.67 SD. A similar criterion has been used in the evaluation of RGW in infancy and early childhood.^26^, ^27^ A gain >0.67 SD may be clinically interpreted as upward centile crossing through at least one of the centile bands in childhood growth charts (e.g., 2nd, 10th, 25th, 50th, 75th, 90th, and 98th centile lines).^26^, ^28^

### 2.6 Metabolic/endocrine measurements

Fasting blood samples were collected at 8 a.m. The concentrations of total cholesterol, high‐density lipoprotein (HDL) and low‐density lipoprotein (LDL) cholesterol, triglycerides, fasting blood glucose, basal insulin, leptin, and ghrelin were determined. Metabolic syndrome (MetS) was defined when ≥3 of the following criteria were met, as used previously^29^: (1) BMI ≥IOTF‐30 for age and sex,^30^ (2) elevated systolic or diastolic blood pressure defined as a value ≥90th percentile for age, sex, and height,^31^ (3) HDL‐C ≤0.4 g/L,^32^ (4) TG triglycerides ≥1.3 g/L,^32^ and (5) homeostasis model assessment of insulin resistance (HOMA‐IR) ≥75th percentile for age and sex.^33^

The levels of thyroid‐stimulating hormone (TSH), serum thyroxine (T4), and serum triiodothyronine (T3) were determined.

Precocious puberty was determined by breast‐tanner stage 2 in girls and testicular volume >4 ml in boys (genital tanner stage 2) and confirmed by the endocrinologist with plasma luteinizing hormone (LH), follicle stimulating hormone (FSH) levels, bone age, and pelvic ultrasound in girls if necessary.

### 2.7 Personal history

Personal history of children regarding birth weight, narcolepsy history, current cataplexy symptoms, hypnagogic hallucinations, sleep paralysis, night eating, academic situation (school difficulties, grade repetition, and absenteeism), and H1N1 vaccination (yes/no answer for all these characteristics) were also collected by the pediatric sleep specialist. The socioeconomical level (SEL) was evaluated based on the parents’ occupational levels.

### 2.8 Follow‐up

Patients were treated following the French recommendations for the management of narcoleptic patients (modafinil, methylphenidate, and sodium oxybate) associated or not with venlafaxine for cataplexy.^34^, ^35^ BMI, BMI z‐score, AESS, and academic situation were collected at the last follow‐up visit. All the patients were treated and followed by the same pediatrician sleep specialist (P.F). Only children for whom the duration of follow‐up was ≥1 year were included in the analyses.

Overall, our primary objective was to compare at the diagnosis time, the narcoleptic symptoms and comorbidities (anthropometry, metabolic, and endocrinal characteristics) between RWG and non‐RWG groups; the second objective was to study at diagnosis the polysomnographic characteristic of these two groups; finally, we evaluated if the pharmacological management and the follow‐up outcomes (clinical and anthropometric changes and academic achievements) were different between these patients.

### 2.9 Statistical analyses

Statistical analyses were conducted using the R software (version 3.6.3, Vienna, Austria).

Continuous variables were expressed as median (range). Dichotomous and polytomous variables were expressed as count (percentage). Comparisons between groups (RWG vs non‐RWG) were performed using the unpaired Wilcoxon tests for continuous variables because of the nonnormality of the distribution assessed by the Shapiro–Wilk test. The Fisher's exact test was used for dichotomous variables, and a *χ*² test was performed for polychotomous variables. The comparisons between disease diagnosis and last follow‐up were conducted using the paired Wilcoxon tests for continuous variables and the Fisher's exact test for dichotomous variables.

A bivariate analysis was conducted and identified factors that were assessed in a multivariable linear model at disease diagnosis, in order to evaluate their association with the BMI z‐score slope reported to one year adjusted for sex (these factors were BMI z‐score at symptom onset, age at diagnosis, and Epworth Sleepiness Scale score at diagnosis). A square root transformation (sqrt) was used to correct the skewed distribution of the slope. For a better readability, figures report BMI z‐score slope per year without square root transformation. Statistical significance value was set to a p‐value <0.05.

## 3 RESULTS

### 3.1 Demographic and general characteristics

**FIGURE 1:**
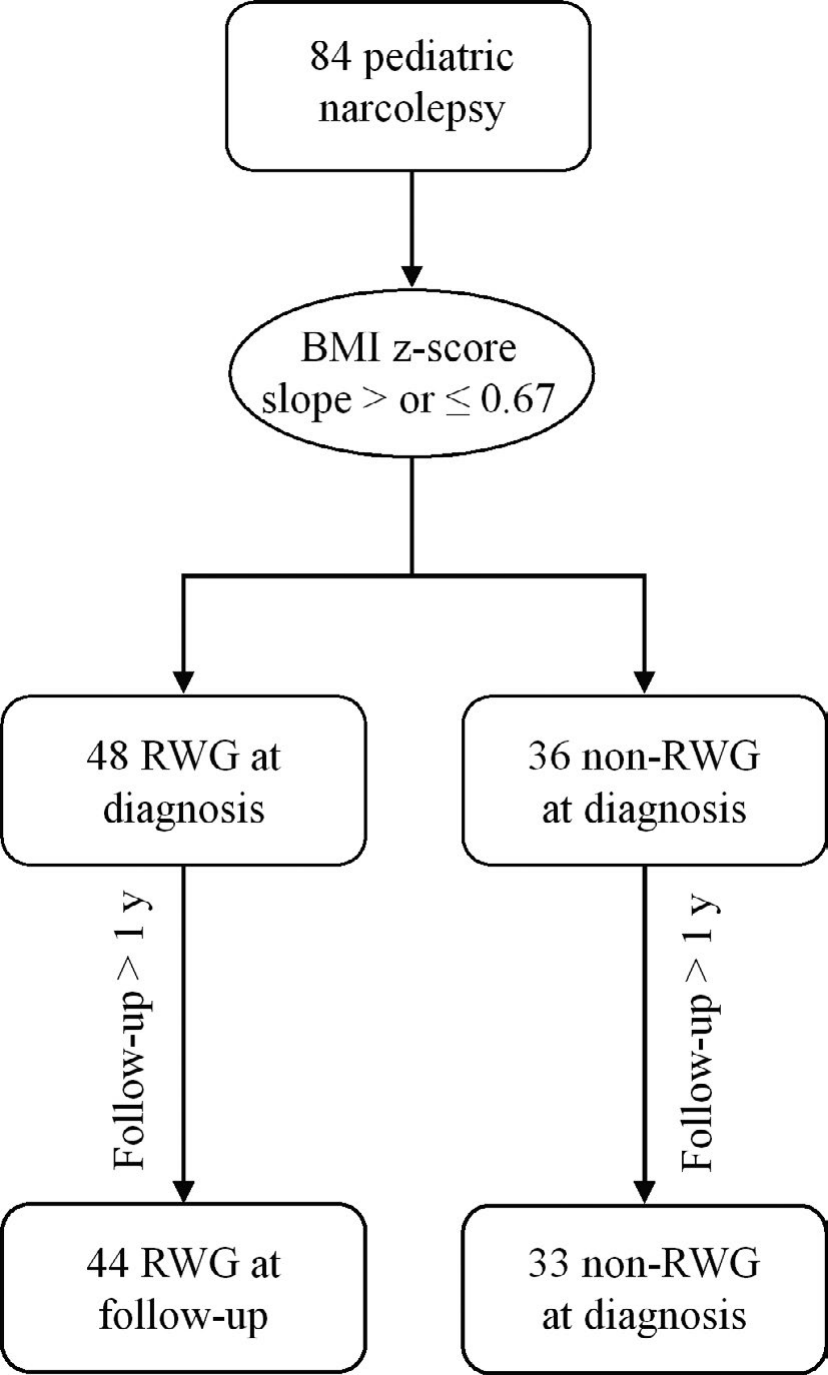
Flowchart in pediatric RWG and non‐RWG narcolepsy. BMI, body mass index; RWG, rapid weight gain

**FIGURE 2:**
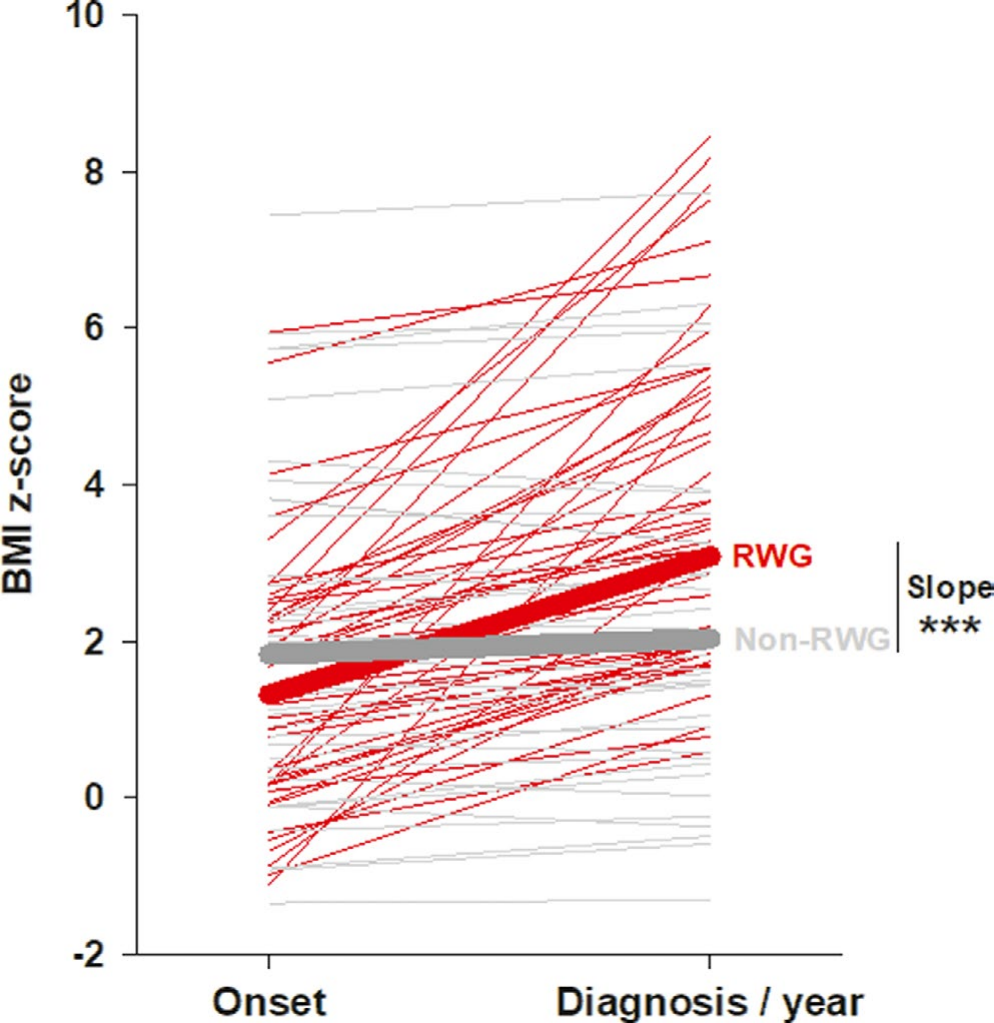
BMI z‐score slope (reported to one year) in pediatric narcoleptic patients with and without rapid weight gain. Each line corresponds to the BMI z‐score slope (reported to one year) of a patient, which is the difference between the BMI z‐score at symptom onset and at disease diagnosis divided by the delay in years between both. The red segments represent RWG patients (slope >0.67), the grey ones represent non‐RWG patients (slope ≤0.67). The red bold line and the grey bold line correspond to the median BMI z‐score slope (reported to one year) of RWG patients (slope =1.8) and non‐RWG patients (slope = 0.2), respectively. The difference of the slope was represented using asterisk (*p < 0.05, **p < 0.01,***p < 0.001). BMI, body mass index; RWG, rapid weight gain

A total of 84 narcoleptic pediatric patients were included in the study; there were 48 patients (57%) in the RWG group and 36 patients (43%) in the non‐RWG group (Table 1; Figure 1). The median (range) BMI z‐score slope was 1.8 (0.7–6.2) for RWG patients and 0.2 (−0.6–0.6) for non‐RGW patients, i.e., significantly higher in the RWG group (*p* < 0.001; Figure 2). There was a tendency for more boys in the RWG than no‐RWG group, but no significant difference was reached. The median age at diagnosis was significantly lower for RWG patients (10.4 vs 12.9 years, *p *= 0.018). Additionally, the median diagnostic delay was shorter in the RWG group (1.1 vs 2.1 years, *p *< 0.001). CSF hypocretin levels were evaluated for 35 patients and were <110 pg/ml for all of them. There was no significant difference in terms of CSF hypocretin concentrations, HLA‐DQB1*06:02 positivity, H1N1 vaccination prior to symptom onset, birth weight, and parents’ socioeconomic levels between RWG and non‐RWG groups (Table 1).

**TABLE 1:** Demographic and general characteristics among narcoleptic patients with and without rapid weight gain (RWG)

	Total	*n*	RWG	N	Non‐RWG	*n*	*p*
Male, *n* (%)	50 (59.5)	84	33 (68.8)	48	17 (47.2)	36	0.072
Age at sleepiness onset, y	10.0 (4.2–15.5)	84	9.0 (4.2–15.5)	48	10.8 (5.6–14.7)	36	0.311
Age at cataplexies onset, y/patients with cataplexy (*n*)	11.0 (4.3–16.6)	65/76	9.8 (4.3–16.6)	36/42	12.0 (6.0–16.6)	29/34	0.539
Age at disease diagnosis, y	12.0 (5.3–17.5)	84	10.4 (5.3–17.5)	48	12.9 (7.4–16.8)	36	0.018
Diagnostic delay, y	1.4 (0.3–5.6)	84	1.1 (0.3–5.0)	48	2.1 (0.5–5.6)	36	<0.001
HLA‐DQB1*06:02 positivity, *n* (%)	76 (98.7)	77	43 (100.0)	43	33 (97.1)	34	0.442
CSF hypocretin−1 concentration, pg/ml	10 (0–90)	35	10 (0–90)	21	21 (1–50)	14	0.830
H1N1 vaccination prior onset, *n* (%)	12 (17.9)	67	5 (13.2)	38	7 (24.1)	29	0.338
Birth weight, g	3.3 (1.9–5.4)	75	3.3 (2.8–5.4)	43	3.3 (1.9–4.1)	32	0.834
Precocious puberty	12 (14.3)	84	5 (10.4)	48	7 (19.4)	36	0.346
SEL of mothers, *n* (%)		65		39		26	0.419
Farmers	3 (4.6)		1 (2.6)		2 (7.7)		
Artisans, shopkeepers	1 (1.5)		0 (0.0)		1 (3.8)		
Executive and intellectual professions	9 (13.8)		6 (15.4)		3 (11.5)		
Intermediate professions	21 (32.3)		12 (30.8)		9 (34.6)		
Employees	20 (30.8)		12 (30.8)		8 (30.8)		
Workers	3 (4.6)		1 (2.6)		2 (7.7)		
Students and pensioners	0 (0.0)		0 (0.0)		0 (0.0)		
Unemployed	8 (12.3)		7 (17.9)		1 (3.8)		
SEL of fathers, *n* (%)		61		39		22	0.542
Farmers	1 (1.6)		1 (2.6)		0 (0.0)		
Artisans, shopkeepers	6 (9.8)		5 (12.8)		1 (4.5)		
Executive and intellectual professions	13 (21.3)		7 (17.9)		6 (27.3)		
Intermediate professions	10 (16.4)		6 (15.4)		4 (18.2)		
Employees	7 (11.5)		5 (12.8)		2 (9.1)		
Workers	16 (26.2)		9 (23.1)		7 (31.8)		
Students and pensioners	1 (1.6)		0 (0.0)		1 (4.5)		
Unemployed	7 (11.5)		6 (15.4)		1 (4.5)		

Note

Data are expressed as median (range) or count (percentage).

Abbreviations: CSF, cerebrospinal fluid; HLA, human leukocyte antigen; RWG, rapid weight gain; SEL, socioeconomical level. The significance level was set at 5%; y, years.

### 3.2 Narcoleptic symptoms and comorbidities

No difference regarding the severity of cataplexy (type 1, 2, 3) or frequency of different types of cataplexy was observed between the two groups (Supplementary Table 1). There was no difference between groups regarding hypnagogic hallucinations and sleep paralysis. Similarly, the frequency of night eating was not significantly different between groups. Compared to non‐RWG patients, narcoleptic patients with RWG had a significantly higher median AESS score (17.0 vs 15.5, *p *= 0.013) and had higher % of patients with AESS score greater than 10 (100.0% vs 86.1%, *p *= 0.012) Additionally, narcoleptic patients with RWG had a higher ISI score (14.5 vs 11.0, *p *= 0.021) and tended to have a higher Conners score (12.5 vs 9.0, *p *= 0.056). The depression and severity of the disease scores were similar between patients with and without RWG (Table 2). There was no significant difference in terms of academic problems (school difficulties, grade repetition, and absenteeism) between both groups (Supplementary Table 2).

**TABLE 2:** Narcoleptic symptoms and comorbidities of narcoleptic patients with and without rapid weight gain (RWG)

	Total	*n*	RWG	*n*	Non‐RWG	*n*	*p*
Cataplexy, *n* (%)	76 (90.5)	84	42 (87.5)	48	34 (94.4)	36	0.457
Hypnagogic hallucinations, *n* (%)	39 (46.4)	84	21 (43.8)	48	18 (50.0)	36	0.660
Sleep paralysis, *n* (%)	17 (20.7)	82	11 (22.9)	48	6 (17.6)	34	0.595
Night eating, *n* (%)	16 (25.8)	62	10 (31.2)	32	6 (20.0)	30	0.390
AESS score	17.0 (6.0–23.0)	84	17.0 (11.0–23.0)	48	15.5 (6.0–23.0)	36	0.013
AESS >10, *n* (%)	79 (94.0)	84	48 (100.0)	48	31 (86.1)	36	0.012
CDI score	11.0 (0.0–38.0)	62	11.0 (2.0–38.0)	36	10.0 (0.0–30.0)	26	0.737
Conners score	10.0 (0.0–23.0)	63	12.5 (0.0–23.0)	38	9.0 (0.0–17.0)	25	0.056
ISI score	13.0 (2.0–22.0)	61	14.5 (2.0–22.0)	38	11.0 (4.0–19.0)	23	0.021
NSS	23.0 (10.0–53.0)	54	22.5 (11.0–53.0)	36	23.0 (10.0–44.0)	18	0.963

Note

Data are expressed as median (range) or count (percentage).

Abbreviation: AESS, adapted Epworth Sleepiness Scale; CDI, Children's Depression Inventory; ISI, Insomnia Severity Index; NSS, Narcolepsy Severity Scale; RWG, rapid weight gain. The significance level was set at 5%.

### 3.3 Anthropometric characteristics

The median BMI z‐score was significantly higher among narcoleptic patients with RWG than that of non‐RWG patients at disease diagnosis (3.5 vs 2.0, *p *= 0.001); however, no significant difference was found at symptom onset (1.3 vs 1.8, *p *= 0.274). The proportion of obese patients was higher among RWG patients at disease diagnosis (75.0 vs 44.4%, *p *= 0.009), but not at symptom onset (25.0 vs 30.6%, *p *= 0.786). No significant difference regarding height delta (height difference between symptom onset and disease diagnosis) was found between groups (Table 3).

**TABLE 3:** Anthropometric characteristics of narcoleptic patients with and without rapid weight gain (RWG)

	Total	*n*	RWG	*n*	Non‐RWG	*n*	*p*
Symptom onset							
BMI z‐score	1.5 (−1.3–7.4)	84	1.3 (−1.1–6.0)	48	1.8 (−1.3–7.4)	36	0.274
BMI classification		84		48		36	0.786
Normal, *n* (%)	36 (42.9)		22 (45.8)		14 (38.9)		
Overweight, *n* (%)	25 (29.8)		14 (29.2)		11 (30.6)		
Obesity, *n* (%)	23 (27.4)		12 (25.0)		11 (30.6)		
Disease diagnosis							
BMI z‐score	3.1 (−1.3–9.0)	84	3.5 (0.5–9.0)	48	2.0 (−1.3–8.6)	36	0.001
BMI classification		84		48		36	0.009
Normal, *n* (%)	15 (17.9)		4 (8.3)		11 (30.6)		
Overweight, *n* (%)	17 (20.2)		8 (16.7)		9 (25.0)		
Obesity, *n* (%)	52 (61.9)		36 (75.0)		16 (44.4)		
Height delta (diagnosis ‐ onset), cm	9.0 (0.0–30.0)	83	5.5 (0.0–30.0)	48	10.0 (0.0–30.0)	35	0.071

Note

Data are expressed as median (range) or count (percentage).

Abbreviation: BMI, body mass index; RWG, rapid weight gain. The significance level was set at 5%.

### 3.4 Metabolic and endocrinal characteristics

There was no difference regarding the total cholesterol, HDL cholesterol, LDL cholesterol, triglycerides, fasting glucose, basal insulin, HOMA, leptin, ghrelin, TSH, T3, and T4 levels between groups. No difference regarding the blood pressure was noted between the two groups. A total of 5/29 (17.2%) RWG narcoleptic children displayed a MetS, whereas none did in the non‐RWG group (*p *= 0.142). No difference in terms of precocious puberty or hypothyroidism was noted between groups (Supplementary Table 3).

### 3.5 Sleep‐related characteristics

No significant difference was noticed between the two groups in terms of nocturnal respiratory parameters and sleep characteristics such as total sleep duration, sleep quality, and alteration of sleep architecture (Table 4). Regarding MSLT, it was evaluated with 5 nap opportunities for 13 patients and with 4 for the remaining 71 patients. There were 3 out of 84 patients who had a mean sleep latency ≥8 min (8, 8.18, 9.4), and all of them presented at least two SOREMPs. Two patients had only one SOREMPs while their mean sleep latency was 4 min (<8 min). There was no significant difference regarding mean sleep latency, REM latencies, and SOREMPs between groups (Table 4).

**TABLE 4:** Sleep‐related characteristics of narcoleptic patients with and without rapid weight gain (RWG)

	Total	*n*	RWG	*n*	Non‐RWG	*n*	*p*
Polysomnography							
TST, min	467 (270 −593)	81	456 (270–593)	45	478 (359–561)	36	0.159
Sleep efficiency, %	84.1 (52.5–95.2)	80	81.6 (52.5–95.2)	45	85.1 (60.8–95.2)	35	0.131
Sleep latency, min	4.3 (0.0–78.0)	80	4.0 (0.0–78.0)	45	5.0 (0.0–30.0)	35	0.763
REM latency, min	5.0 (0.0–225.0)	79	9.0 (0.0–225.0)	45	4.0 (0.0–211.0)	34	0.510
N1 sleep, % TST	14.3 (0.2–35.8)	77	14.3 (2.7–35.8)	44	14.1 (0.2–28.3)	33	1.000
N2 sleep, % TST	40.4 (16.1–64.7)	77	40.4 (16.1–64.7)	44	40.4 (19.8–58.3)	33	0.853
N3 sleep, % TST	20.6 (7.4–59.5)	80	20.6 (9.1–59.5)	45	20.8 (7.4–43.0)	35	0.705
REM sleep, % TST	22.4 (10.5–39.3)	80	22.3 (10.5–39.3)	45	22.9 (10.6–32.6)	35	0.528
AHI, *n*/h	0.6 (0.0–11.9)	81	0.6 (0.0–11.9)	45	0.6 (0.0–9.4)	36	0.841
Minimal oxygen saturation, %	92.8 (30.0–98.0)	71	93.0 (74.0–98.0)	41	92.6 (30.0–97.0)	30	0.567
Multiple sleep latency test							
N 5 test, *n* (%)	13 (15.5)	84	4 (8.3)	48	9 (25.0)	36	0.065
Mean sleep latency, min	2.7 (0.3–9.4)	84	2.3 (0.3–9.4)	48	3.4 (0.5–7.8)	36	0.619
REM sleep latency, min	4.5 (1.0–14.3)	68	4.0 (1.0–11.0)	40	5.3 (1.5–14.3)	28	0.170
SOREMPs, *n*	4 (1–5)	84	4 (2–4)	48	4 (1–5)	36	0.686
SOREMPs, %	100 (25–100)	84	100 (40–100)	48	80 (25–100)	36	0.209

Note

Data are expressed as median (range) or count (percentage).

Abbreviation: AHI, apnea–hypopnea index; REM, rapid eye movement; RWG, rapid weight gain; SOREMPs, sleep onset rapid eye movement periods; TST, total sleep time. The significance level was set at 5%.

### 3.6 Follow‐up

**FIGURE 3:**
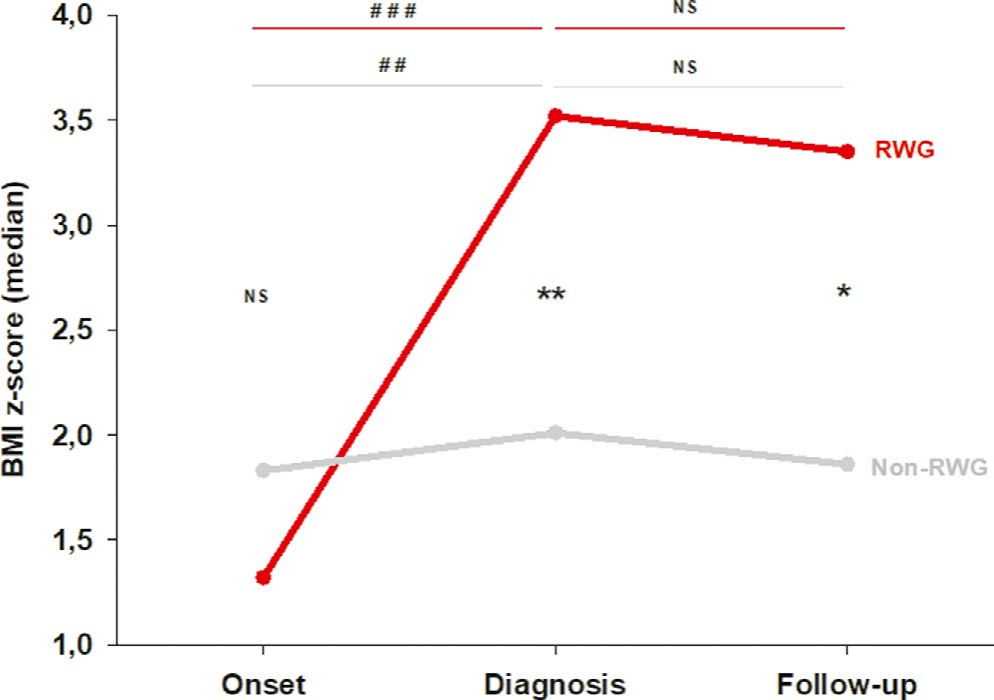
BMI z‐score progression over time in pediatric narcoleptic children with and without rapid weight gain The red line represents the BMI z‐score progression in RWG patients, three red points represent the median BMI z‐score at symptom onset, disease diagnosis, and last follow‐up of RWG patients, respectively; the grey line represents the BMI z‐score progression in non‐RWG patients, three grey points represent the median BMI z‐score at symptom onset, disease diagnosis, and last follow‐up of non‐RWG patients, respectively. The difference between RWG patients and non‐RWG patients was presented using asterisk (*p < 0.05, **p < 0.01, ***p < 0.001) at symptom onset, disease diagnosis, and follow‐up, respectively. The difference between symptom onset, disease diagnosis, and follow‐up was presented using hashtag (# *p *< 0.05, # # *p *< 0.01, # # # *p *< 0.001) within RWG patients and within non‐RWG patients, respectively. BMI, body mass index; RWG, rapid weight gain

No follow‐up after diagnosis occurred for 4 patients, and 3 patients were excluded as their follow‐up lasted less than 1 year. Most narcoleptic children were followed‐up until they were 18‐year‐old by the same pediatrician sleep specialist (P.F.) they consulted at diagnosis (Figure 1). The age at the last follow‐up visit was similar between groups. The follow‐up duration was longer for RWG patients than that for non‐RWG patients due to the earlier disease diagnosis among patients with RWG. At last follow‐up, narcoleptic children with RWG still had a higher BMI z‐score (3.4 vs 1.9, *p *= 0.011; Figure 3) and were more frequently obese (50.0% vs 21.2%, *p *= 0.033) compared to those with non‐RWG; however, no difference regarding the Epworth score and school difficulties was noted between the two groups. After accounting for the duration of follow‐up (normalization), no difference between groups was found regarding the total number of treatments administered. No difference in terms of number of patients administered levothyroxine, sodium oxybate, or anticataplectic treatments was noted between RWG and non‐RWG patients. The total number of treatments and the number of patients receiving anti‐cataplectic treatments at last follow‐up visit was similar between the two groups of patients (Table 5).

**TABLE 5:** Follow‐up of narcoleptic patients with and without rapid weight gain (RWG)

	Total	*n*	RWG	*N*	Non‐RWG	*n*	*p*
Follow‐up duration, years	5.1 (1.2–11.4)	77	5.4 (2.2–11.4)	44	4.4 (1.2–9.1)	33	0.016
Age, years	17.4 (8.0–19.7)	77	17.4 (8.0–19.7)	44	17.4 (13.3–18.7)	33	0.955
BMI	24.2 (16.2–49.5)	77	25.6 (16.2–49.5)	44	23.1 (17.6–40.9)	33	0.023
BMI z‐score	2.4 (−0.8–12.7)	77	3.4 (−0.6–12.7)	44	1.9 (−0.8–10.3)	33	0.011
BMI classification		77		44		33	0.033
Normal, *n* (%)	29 (37.7)		14 (31.8)		15 (45.5)		
Overweight, *n* (%)	19 (24.7)		8 (18.2)		11 (33.3)		
Obesity, *n* (%)	29 (37.7)		22 (50.0)		7 (21.2)		
Epworth score	8 (1–19)	77	8 (3–17)	44	9 (1–19)	33	0.951
School difficulties	19 (24.7)	77	13 (29.5)	44	6 (18.2)	33	0.295
Treatments							
Total number of treatments	4 (1–8)	77	4 (1–8)	44	3 (1–7)	33	0.017
Total number of treatments/year	0.7 (0.1–4.1)	77	0.7 (0.2–2.1)	44	0.7 (0.1–4.1)	33	0.963
Number of patients who received anticataplectic treatments	26 (33.8)	77	14 (31.8)	44	12 (36.4)	33	0.808
Number of patients who received sodium oxybate	31 (40.3)	77	19 (43.2)	44	12(36.4)	33	0.641
Number of patients who received levothyroxine	4 (5.2)	77	2 (4.5)	44	2 (6.1)	33	1.000
Total number of treatments at the last visit	2 (1–6)	77	2 (1–6)	44	2 (1–4)	33	0.392
Number of patients with anticataplectic at the last visit	16 (20.8)	77	7 (15.9)	44	9 (27.3)	33	0.264

Note

Data are expressed as median (range) or count (percentage).

Abbreviation: BMI, body mass index; RWG, rapid weight gain. The significance level was set at 5%.

There was no difference in BMI z‐score between diagnosis and last follow‐up visit for neither RWG (3.6 vs 3.4, *p *= 0.695) nor non‐RWG (2.0 vs 1.9, *p *= 0.685) patients. However, the proportion of obese patients was significantly lower among RWG patients at the last visit compared to the time of diagnosis (50.0% vs 77.3%, *p *= 0.008), whereas this difference was not significant for narcoleptic patients with non‐RWG (21.2% vs 42.4%, *p *= 0.171). At the last follow‐up visit, both RWG and non‐RWG groups had a significantly lower Epworth score (8 vs 18, *p *< 0.001 and 9 vs 15, *p* < 0.001, respectively), lower % patients with an Epworth score >10 (34.1% vs 100.0%, *p *< 0.001 and 45.5% vs 84.8%, *p *= 0.002, respectively) or with school difficulties (29.5% vs 53.5%, *p *= 0.030 and 18.2% vs 50.0%, *p *= 0.015, respectively) compared to the time of disease diagnosis (Table 6).

**TABLE 6:** Comparison between diagnosis and last follow‐up of narcoleptic patients with and without rapid weight gain (RWG)

	Diagnosis	*n*	Last follow‐up	*n*	*p*
RWG patients					
BMI	22.9 (16.1–31.6)	44	25.6 (16.2–49.5)	44	<0.001
BMI z‐score	3.6 (0.5–9.0)	44	3.4 (−0.6–12.7)	44	0.695
BMI classification		44		44	0.008
Normal	3 (6.8)		14 (31.8)		
Obesity	34 (77.3)		22 (50.0)		
Overweight	7 (15.9)		8 (18.2)		
Epworth score	18 (11–23)	44	8 (3–17)	44	<0.001
Epworth >10	44 (100.0)	44	15 (34.1)	44	<0.001
School difficulties	23 (53.5)	43	13 (29.5)	44	0.030
Non‐RWG patients					
BMI	21.5 (15.9–31.6)	33	23.1 (17.6–40.9)	33	0.002
BMI z‐score	2.0 (−0.5–6.3)	33	1.9 (−0.8–10.3)	33	0.685
BMI classification		33		33	0.171
Normal	10 (30.3)		15 (45.5)		
Obesity	14 (42.4)		7 (21.2)		
Overweight	9 (27.3)		11 (33.3)		
Epworth score	15 (6–23)	33	9 (1–19)	33	<0.001
Epworth >10	28 (84.8)	33	15 (45.5)	33	0.002
School difficulties	15 (50.0)	30	6 (18.2)	33	0.015

Note

Data are expressed as median (range) or count (percentage).

Abbreviation: BMI, body mass index; RWG, rapid weight gain. The significance level was set at 5%.

### 3.7 Multivariable regression

**FIGURE 4:**
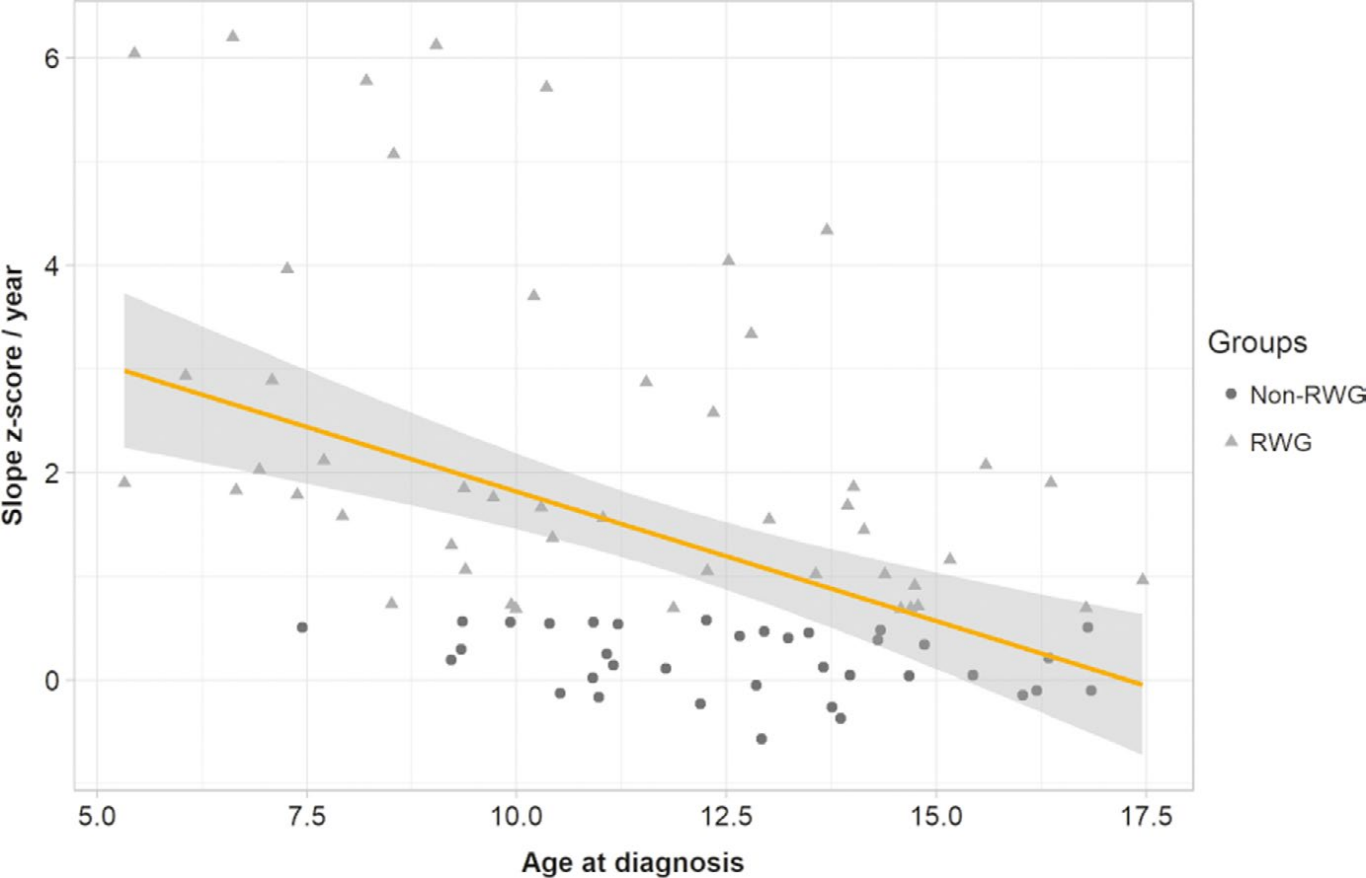
BMI z‐score slope (reported to one year) according to age at disease diagnosis in pediatric narcoleptic patients. Each point represents one patient: triangles for RWG narcoleptic children and circles for non‐RGW children. A significant decrease of the BMI z‐score slope was observed with the increase of the age at diagnosis. RWG, rapid weight gain

**FIGURE 5:**
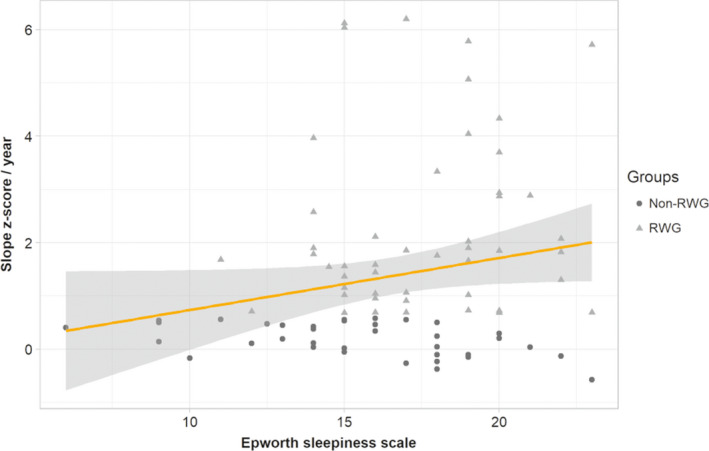
BMI z‐score slope (reported to one year) according to AESS score in pediatric narcoleptic patients. Each point represents one patient: triangles for RWG narcoleptic children and circles for non‐RGW children. A significant increase of the BMI z‐score slope was observed with the increase of the AESS score. RWG, rapid weight gain

The adjusted analysis, explaining 28% of the BMI z‐score slope per year variability, found significant independent associations between the BMI z‐score slope per year and the age at disease diagnosis (*p *< 0.001, Figure 4) and the Epworth Sleepiness Scale at diagnosis (*p *= 0.003, Figure 5). The higher the increase of BMI z‐score slope, the lower the age at diagnosis and the worse the Epworth Sleepiness Score at diagnosis. There was no other significant effect.

## 4 DISCUSSION

**FIGURE 6:**
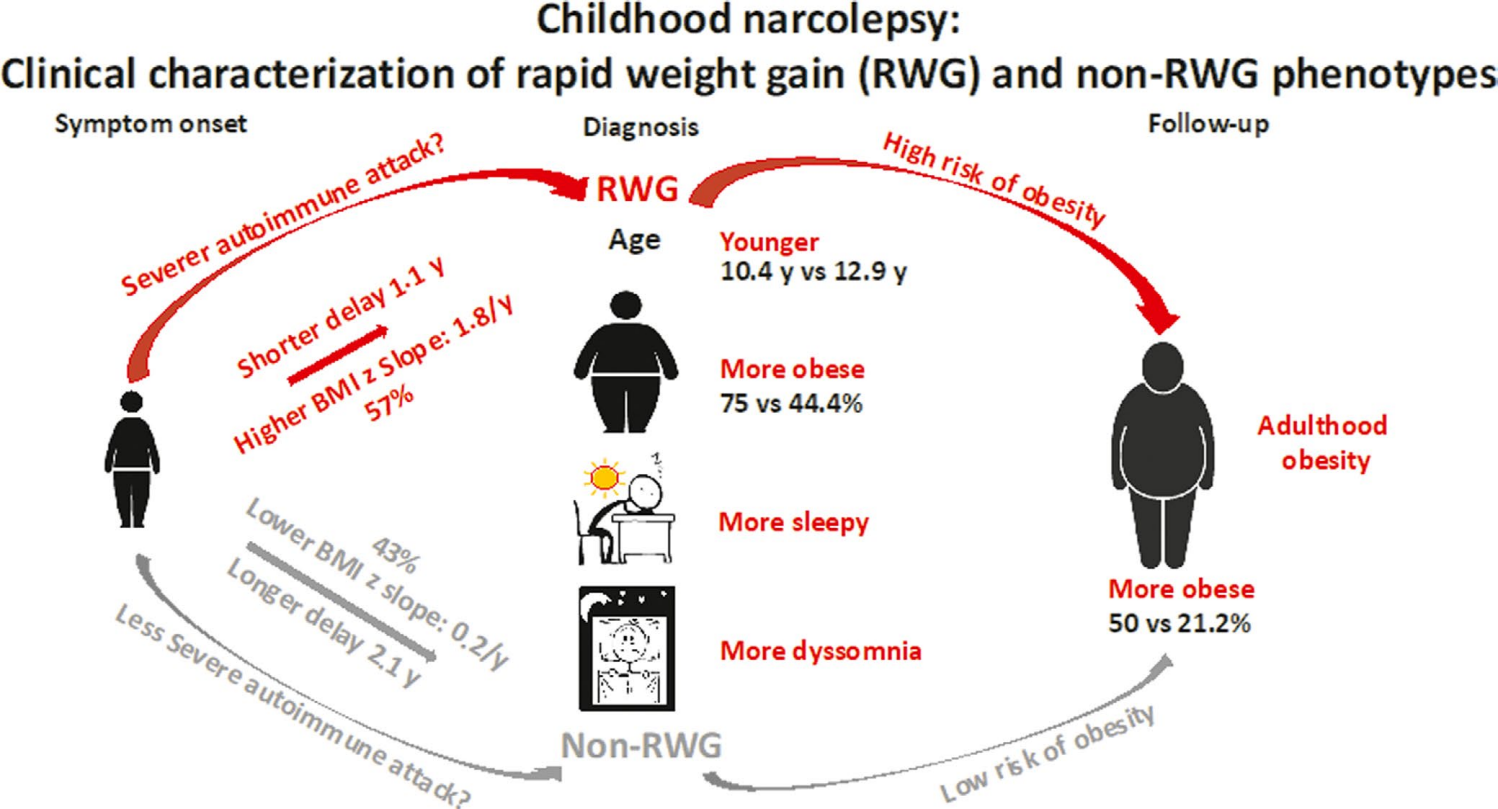
Characterization of rapid weight gain (RWG) and non‐RWG phenotype of childhood narcolepsy. Compared to non‐RWG patients, RWG narcoleptic children had shorter diagnosis delay, were younger, more obese, sleepier with more dyssomnia at diagnosis, but were also more obese at follow‐up. RGW could represent a maker of a more severe phenotype of childhood narcolepsy. BMI, body mass index; RWG, rapid weight gain

Compared to non‐RWG patients, despite a shorter diagnosis delay, RWG narcoleptic patients were younger, sleepier, and their prevalence of obesity was higher at disease diagnosis but not at symptom onset, which is indicative of a more accelerated pathological process. At last follow‐up, both groups displayed improvement in sleepiness and school difficulties; however, RWG patients still had a higher BMI z‐score and a higher prevalence of obesity compared to that of non‐RWG patients, despite in the RWG group, a decrease in the proportion of obese patients compared to disease diagnosis (Figure 6).

Studies investigating the prevalence and the pathophysiology of obesity in narcoleptic adults remain controversial.^36^, ^37^ An early age at disease diagnosis seems to increase the susceptibility of developing obesity.^8^, ^12^ Due to the fast growth, metabolism, and weight changes occurring during childhood and adolescence, the loss of hypocretin might yield exaggerated weight gain, perhaps due to relatively decreased activities and metabolic consumption.^13^ Additionally, an impairment in histamine neurotransmission, from another brain wake‐promoting system located in close proximity to hypocretin neurons, has been reported in the CSF of NT1 pediatric patients,^38^ but not in those of adults.^39^ Histaminergic neurons could also be involved in the rapid weight gain process as a mild obesity has been observed in knockout mice lacking either histamine or hypocretins.^40^, ^41^ While the CSF histamine levels remained to be determined in RWG and non‐RWG patients, there was no significant difference regarding the CSF hypocretin levels between groups herein, likely due to the small number of patients involved. Moreover, various evidence from animal models and adult patients have supported the idea that both a decreased metabolism and subtle changes in eating behavior (rather than in calorie intake) are responsible for the positive energy balance leading to obesity in the context of narcolepsy.^26^, ^30^ However, in the present study, no difference regarding night‐eating behaviors was found between groups.

Nonetheless, as a consequence of maturation, children need more sleep, particularly nonrapid eye movement (NREM) sleep. RWG patients had a higher insomnia severity index score and daytime sleepiness, suggesting that they experienced severer sleep disturbance compared to non‐RWG patients. Interestingly, poor quality of sleep has been reported in overweighed children and adolescents even without sleep breathing disturbances,^42^ perhaps due to endocrine alterations such as decreased levels of leptin, increased levels of ghrelin, and compromised insulin sensitivity.^43^ However, we did not find significant differences in terms of sleep duration, sleep quality, or alteration of sleep architecture between groups with this limited sample.

On the other hand, obesity could be also considered as a risk factor for sleep disturbance, as an increased BMI has been associated with a higher risk of obstructive sleep apnea (OSA).^44^ Obstructive sleep breathing may result in frequent awakenings and altered sleep quality and duration,^44^ even if we found no significant difference in respiratory parameters.

The shorter delay between symptom onset and disease diagnosis argues for a faster disease progression. The RWG in narcoleptic children could be a marker of severity of the disease, with more pronounced symptoms at onset that could reflect a more invasive autoimmune‐inflammatory attack. Indeed, autoimmune processes could play a determinant role in the pathophysiology of narcolepsy;^6^, ^45^, ^46^ H1N1 influenza or streptococcus infections could stimulate autoreactive T‐cells or B‐cells, then an enhanced permeability of the blood–brain barrier provoked by fever and inflammation could facilitate autoreactive T‐cell transfer into the brain that may induce a transient autoimmune attack of hypocretin cells.^47^ In addition, interactions between sleep and innate immunity are considered as bidirectional, as immune activation alters sleep, and sleep disturbance in turn could affect immune functions.^48^ Until now, immunotherapies in narcoleptic patients have reported poor beneficial results.^49^ However, immunotherapy intervention in targeted patients such as RWG children could give the opportunity to stop the autoimmune process and thus reduce the hypocretin neuronal loss at the early stages of the disease. More studies are required to understand the underlying pathophysiological mechanisms related to RWG.

This RWG phenotype could also have long‐term repercussions such as a high risk of obesity at adulthood. Obesity is a burden for their future adult life and is associated with a series of potential complications such as cardiovascular diseases, metabolic syndrome, diabetes, and poor quality of life.^50^ Although during follow‐up these children benefited from multidisciplinary management for obesity with endocrinologists, nutritionists, physical therapists, and psychologists and although their daytime sleepiness had been significantly improved after therapeutic management, the group of RWG patients still had at last follow‐up a higher BMI z‐score and a higher prevalence of obesity. Sodium oxybate has been showed to improve sleepiness, frequency of cataplexy, and sleep quality,^34^, ^35^ but also to prevent weight gain in children.^12^, ^51^ Thus, European guidelines and expert statement on the management of narcolepsy in 2021 recommended that sodium oxybate should be proposed to narcoleptic children, especially those with obesity. In France, sodium oxybate received the agreement of the public health for narcoleptic children only during the summer 2021. Since then, patients and parents were often reluctant to accept this treatment.

The present study suffers from several limitations. First, we could assume that these results concerned NT1 children. However, not all children with narcolepsy had cataplexy and/or CSF hypocretin level determined. CSF hypocretin concentrations were measured in less than half of the included patients. Consequently, this number was insufficient to assess the role of hypocretins in metabolism. However, all the tested patients were hypocretin‐deficient (<110 pg/ml) and a majority of patients presented cataplexy except for 8 patients, and among them 3 had low CSF hypocretin levels. Second, this is a retrospective study, the BMI z‐score was determined only at symptom onset and at disease diagnosis, and there were no consecutive data available regarding weight and height between these two time points, or before symptom onset. The definitions of rapid growth are various among published studies. Variations greater than +0.67 SD in weight‐for‐age z‐scores between two separate evaluations have been used in previous studies,^28^, ^52^ as well as variations greater than +0.67 SD in weight‐for‐age, height‐for‐age, and weight‐for‐height z‐scores.^53^ Using weight‐for‐age variation greater than +1 SD z‐score has also been reported to assess rapid growth,^54^ and did not change significantly the results presented here, except than the RWG children were also younger at disease onset. Moreover, to understand the mechanisms involved in the RWG phenotype, some data such as genetic, antenatal, and postnatal environmental risk factors,^28^, ^53^ but also serum and CSF proinflammatory cytokine levels and autoreactive CD8^+^/CD4^+^ T‐cells at symptom onset were missing.^45^, ^46^, ^55^

In conclusion, despite a similar BMI z‐score at symptom onset and a shorter diagnostic delay, narcoleptic pediatric patients with RWG were younger, sleepier, and more obese in proportion than non‐RWG patients. Given that the autoimmune etiology of narcolepsy plays a critical role in its pathophysiology, RGW in narcoleptic children could reflect severer autoimmune‐inflammatory attacks and could inspire the design of specific treatments such as immunological therapy in these patients.^56^, ^57^ RWG could represent a more severe phenotype of the disease with a long‐term persistence of obesity at follow‐up. As the persistence of obesity could represent a risk of developing metabolic and cardiorespiratory complications and poor quality of life, we recommend to assess the growth curve of the narcoleptic children to determine if they display a RWG profile, and to ensure a prompt and more offensive management to prevent obesity and its complications.

## CONFLICT OF INTEREST

The authors declare no conflict of interest.

## AUTHOR CONTRIBUTIONS

P.F., M.Z., M.T., A.G., S.P., and CO.I. designed the study. P.F., CO.I., and N.V. collected the data. S.P. and M.T. conducted the statistical analysis. P.F., M.Z., M.T., A.G., JS.L., S.P., L.A., and C.V. interpreted the results. P.F. and M.Z. drafted the manuscript. P.F., M.Z., M.T., A.G., JS.L., S.P., L.A., and C.V. contributed to the manuscript revision.

## Supporting information

Table S1‐S3

## Data Availability

Data available upon request to the authors.
