# Supplementary material for: Characterization of rapid weight gain phenotype in children with narcolepsy
Source: CNS Neurosci Ther. 2022 Feb 25;28(6):829–41. doi: 10.1111/cns.13811 (PMC9062543; doi:10.1111/cns.13811)
Supplement: Supplementary file 1 — Table S1‐S3 [file CNS-28-829-s001.docx]

**Supplementary table 1. Cataplexy characteristics of narcoleptic patients with and without rapid weight gain (RWG)**

|  | Total | n=76 | RWG | n=42 | Non-RWG | n=34 | p |
| --- | --- | --- | --- | --- | --- | --- | --- |
| Location |  |  |  |  |  |  |  |
| Neck, n (%) | 53 (80.3) | 66 | 31 (77.5) | 40 | 22 (84.6) | 26 | 0.543 |
| Face, n (%) | 51 (76.1) | 67 | 31 (77.5) | 40 | 20 (74.1) | 27 | 0.777 |
| Superior limbs, n (%) | 45 (68.2) | 66 | 27 (67.5) | 40 | 18 (69.2) | 26 | 1.000 |
| Inferior limbs, n (%) | 55 (82.1) | 67 | 31 (77.5) | 40 | 24 (88.9) | 27 | 0.335 |
| Frequency |  |  |  |  |  |  |  |
| Type 1, n (%) | 65 (91.5) | 71 | 36 (87.8) | 41 | 29 (96.7) | 30 | 0.390 |
| Type 1, frequency, n (%) |  | 57 |  | 31 |  | 26 | 0.200 |
| 4 | 28 (49.1) |  | 14 (45.2) |  | 14 (53.8) |  |  |
| 3 | 18 (31.6) |  | 8 (25.8) |  | 10 (38.5) |  |  |
| 2 | 10 (17.5) |  | 8 (25.8) |  | 2 (7.7) |  |  |
| 1 | 1 (1.8) |  | 1 (3.2) |  | 0 (0.0) |  |  |
| 0 | 0 (0.0) |  | 0 (0.0) |  | 0 (0.0) |  |  |
| Type 2, n (%) | 65 (92.9) | 70 | 35 (87.5) | 40 | 30 (100.0) | 30 | 0.066 |
| Type 2, frequency, n (%) |  | 59 |  | 32 |  | 27 | 0.097 |
| 4 | 25 (42.4) |  | 13 (40.6) |  | 12 (44.4) |  |  |
| 3 | 19 (32.2) |  | 8 (25.0) |  | 11 (40.7) |  |  |
| 2 | 12 (20.3) |  | 10 (31.2) |  | 2 (7.4) |  |  |
| 1 | 3 (5.1) |  | 1 (3.1) |  | 2 (7.4) |  |  |
| 0 | 0 (0.0) |  | 0 (0.0) |  | 0 (0.0) |  |  |
| Type 3, n (%) | 34 (50.0) | 68 | 19 (47.5) | 40 | 15 (53.6) | 28 | 0.806 |
| Type 3, frequency, n (%) |  | 31 |  | 16 |  | 15 | 0.543 |
| 4 | 4 (12.9) |  | 3 (18.8) |  | 1 (6.7) |  |  |
| 3 | 9 (29.0) |  | 6 (37.5) |  | 3 (20.0) |  |  |
| 2 | 12 (38.7) |  | 5 (31.2) |  | 7 (46.7) |  |  |
| 1 | 2 (6.5) |  | 1 (6.2) |  | 1 (6.7) |  |  |
| 0 | 4 (12.9) |  | 1 (6.2) |  | 3 (20.0) |  |  |

Data are expressed as median (range) or count (percentage).

The significance level was set at 5%.

**Supplementary table 2. Academic characteristics of narcoleptic patients with and without rapid weight gain (RWG)**

|  | Total | n | RWG | n | Non-RWG | n | p |
| --- | --- | --- | --- | --- | --- | --- | --- |
| Grade repetition, n (%) | 22 (27.8) | 79 | 14 (31.1) | 45 | 8 (23.5) | 34 | 1.000 |
| School difficulties, n (%) | 43 (53.8) | 80 | 27 (57.4) | 47 | 16 (48.5) | 33 | 0.634 |
| Absenteeism, n (%) | 14 (18.2) | 77 | 10 (22.7) | 44 | 4 (12.1) | 33 | 0.360 |

Data are expressed as count (percentage).

The significance level was set at 5%.

**Supplementary table 3. Metabolic and endocrinal characteristics of narcoleptic patients with and without rapid weight gain (RWG)**

|  | Total | n | RWG | n | Non-RWG | n | p |
| --- | --- | --- | --- | --- | --- | --- | --- |
| Total cholesterol, mmol/L | 4.4 (2.1-6.9) | 60 | 4.4 (2.7-6.9) | 36 | 4.5 (2.1-5.4) | 24 | 0.608 |
| HDL cholesterol, mmol/L | 1.2 (0.7-2.0) | 60 | 1.2 (0.8-2.0) | 36 | 1.3 (0.7-1.8) | 24 | 0.192 |
| LDL cholesterol, mmol/L | 2.7 (1.2-4.9) | 58 | 2.8 (1.6-4.9) | 35 | 2.7 (1.2-3.5) | 23 | 0.994 |
| Triglycerides, mmol/L | 0.9 (0.4-4.7) | 61 | 0.9 (0.4-2.7) | 36 | 0.9 (0.4-4.7) | 25 | 0.752 |
| Glucose, mmol/L | 4.9 (1.1-7.0) | 76 | 4.9 (1.1-5.7) | 44 | 4.9 (3.8-7.0) | 32 | 0.879 |
| Insulin, mUI/L | 11.0 (1.0-34.0) | 49 | 10.5 (1.0-34.0) | 30 | 12.0 (7.0-33.0) | 19 | 0.503 |
| HOMA-IR | 2.4 (0.2-8.3) | 49 | 2.3 (0.2-8.3) | 30 | 2.6 (1.4-7.1) | 19 | 0.659 |
| Leptin, ng/mL | 16.6 (0.9-77.4) | 51 | 17.9 (0.9-56.1) | 30 | 13.8 (1.3-77.4) | 21 | 0.781 |
| Total ghrelin, pg/mL | 269.0 (62.0-1283.0) | 43 | 260.0 (62.0-1283.0) | 25 | 294.5 (106.0-623.0) | 18 | 1.000 |
| TSH, mUI/L | 1.5 (0.3-4.8) | 68 | 1.6 (0.3-2.5) | 38 | 1.5 (0.7-4.8) | 30 | 0.892 |
| T3, pg/L | 4.8 (2.9-6.6) | 61 | 4.8 (2.9-6.6) | 36 | 4.8 (3.3-5.9) | 25 | 0.542 |
| T4, pg/L | 13.6 (10.9-20.6) | 66 | 13.9 (10.9-19.9) | 38 | 13.3 (11.3-20.6) | 28 | 0.399 |
| SBP | 82.5 (63.8-116.0) | 69 | 82.5 (63.8-108.0) | 41 | 82.8 (75.0-116.0) | 28 | 0.315 |
| DBP | 45.0 (30.0-68.0) | 69 | 45.0 (30.0-68.0) | 41 | 45.0 (37.5-64.0) | 28 | 0.205 |
| BMI >IOTF30 | 19 (36.5) | 52 | 16 (59.2) | 27 | 3 (12.0) | 25 | 0.036 |
| Metabolic syndrome | 5 (10.4) | 48 | 5 (17.2) | 29 | 0 (0.0) | 19 | 0.142 |
| Precocious Puberty | 12 (14.3) | 84 | 5 (10.4) | 48 | 7 (19.4) | 36 | 0.346 |
| Hypothyroidism | 4 (5.9) | 68 | 2 (5.1) | 39 | 2 (6.9) | 29 | 1.000 |

Data are expressed as median (range) or count (percentage).

Abbreviation: BMI = body mass index; DBP = dystolic blood pressure; HDL = high-density lipoprotein; HOMA-IR = homeostasis model assessment of insulin resistance; LDL = low-density lipoprotein; RWG = rapid weight gain; SBP = systolic blood pressure; TSH = thyroid-stimulating hormone; T3 = serum triiodothyronine; T4 = serum thyroxine. The significance level was chosen at 5%.
